# Building an adverse outcome pathway network for estrogen-, androgen- and steroidogenesis-mediated reproductive toxicity

**DOI:** 10.3389/ftox.2024.1357717

**Published:** 2024-03-26

**Authors:** Johanna Zilliacus, Monica K. Draskau, Hanna K. L. Johansson, Terje Svingen, Anna Beronius

**Affiliations:** ^1^ Institute of Environmental Medicine, Karolinska Institutet, Stockholm, Sweden; ^2^ National Food Institute, Technical University of Denmark, Kongens Lyngby, Denmark

**Keywords:** AOP network, endocrine disruption, ED assessment, reproductive toxicity, EAS modalities, next generation risk assessment

## Abstract

**Introduction:** Adverse Outcome Pathways (AOPs) can support both testing and assessment of endocrine disruptors (EDs). There is, however, a need for further development of the AOP framework to improve its applicability in a regulatory context. Here we have inventoried the AOP-wiki to identify all existing AOPs related to mammalian reproductive toxicity arising from disruption to the estrogen, androgen, and steroidogenesis modalities. Core key events (KEs) shared between relevant AOPs were also identified to aid in further AOP network (AOPN) development.

**Methods:** A systematic approach using two different methods was applied to screen and search the entire AOP-wiki library. An AOPN was visualized using Cytoscape. Manual refinement was performed to remove AOPS devoid of any KEs and/or KERs.

**Results:** Fifty-eight AOPs relevant for mammalian reproductive toxicity were originally identified, with 42 AOPs included in the final AOPN. Several of the KEs and KE relationships (KERs) described similar events and were thus merged to optimize AOPN construction. Sixteen sub-networks related to effects on hormone levels or hormone activity, cancer outcomes, male and female reproductive systems, and overall effects on fertility and reproduction were identified within the AOPN. Twenty-six KEs and 11 KERs were identified as core blocks of knowledge in the AOPN, of which 19 core KEs are already included as parameters in current OECD and US EPA test guidelines.

**Discussion:** The AOPN highlights knowledge gaps that can be targeted for further development of a more complete AOPN that can support the identification and assessment of EDs.

## 1 Introduction

Minimizing potential human health risks from endocrine disruptors (EDs) has been a highly prioritized issue in the European Union (EU) for several years (European Parliament, 2013, 2019; European Commission, 2018b) and is specifically highlighted in the European Chemicals strategy for sustainability (European Commission, 2020). EDs are exogenous substances that interfere with the normal function of the endocrine system to cause adverse health effects in an intact organism, or its progeny, or in (sub)populations (WHO/IPCS, 2002). Since the endocrine system regulates tissue and organ development and function, disruption can lead to a range of serious health effects, including reproductive, developmental, and metabolic disorders, cardiovascular diseases, and cancers (WHO/UNEP, 2013; Gore et al., 2015; Duh-Leong et al., 2023). Thus, endocrine disruption represents a mode of action (MoA) that can lead to various toxicities. EDs exert their toxicity through mechanisms such as direct interference with hormone receptors, as well as interference with hormone synthesis, transport, or metabolism. While the endocrine system comprises many different hormones, signalling pathways and organ systems, regulatory assessment of EDs in the EU currently focuses on substances that interact with the estrogen, androgen, thyroid or steroidogenesis (EATS) modalities. The reason is that there is relatively good mechanistic understanding of how perturbations of the EATS modalities may result in adverse effects and which types of effects may arise (ECHA/EFSA/JRC, 2018). There are also several standardized *in vivo* and *in vitro* test guidelines available from the OECD and US Environmental Protection Agency (EPA) that include relevant endpoints to investigate EATS-related mechanisms and EATS-mediated adverse effects.

Scientific criteria for identifying EDs have been implemented for biocides and plant protection products in the EU (European Commission, 2017; 2018a) and recently in the European legislation for Classification Labelling and Packaging (CLP) (European Commission, 2023). These criteria stipulate that a substance should be considered an ED if: 1) it shows an adverse effect in an intact organism or its progeny, 2) it has an endocrine MoA, and 3) the adverse effect is a consequence of the endocrine MoA. Consequently, identification of EDs requires a high degree of mechanistic understanding, including understanding of the links between early mechanisms and adverse health effects, to establish an endocrine MoA and perform a MoA analysis (ECHA/EFSA/JRC, 2018). The Adverse Outcome Pathway (AOP) framework is particularly well suited for this purpose, as it provides information on all three criteria.

The AOP framework provides a harmonized approach to describe a causal chain of events linking a molecular initiating event (MIE) to key events (KE) at different levels of biological organization, ultimately leading to an adverse outcome (AO) through defined key event relationships (KER) (Ankley et al., 2010; Villeneuve et al., 2014; Ankley and Edwards, 2018). AOPs thus provide a structured format to summarise available evidence for, and confidence in, the causal links between MIEs and AOs; i.e., they are valuable tools for MoA analysis in ED assessment and for developing Integrated Approaches to Testing and Assessment (IATA) (Browne et al., 2017; OECD, 2017; Audouze et al., 2021; Svingen et al., 2022; Bajard et al., 2023; Wiklund et al., 2023). Development of AOPs is currently overseen by the OECD’s Advisory Group on Emerging Science in Chemicals Assessment (ESCA) in collaboration with the Society for the Advancement of AOPs (SAAOP) and AOP Knowledge Base Coordination Group. Fully developed AOPs can be endorsed by the OECD Working Party on Hazard Assessment (WPHA) and/or Working Group of the National Coordinators for the Test Guidelines Program (WNT) after technical review according to a process described in the AOP Developers’ Handbook (OECD, 2023). For the AOPs to be endorsable, it is important that the peer review is overseen either by the OECD AOP Development Programme or by a scientific journal that has a written agreement with the OECD to handle AOP Reports. With the latter option, an acceptance of the journal submission would make it automatically eligible to be considered for endorsement by the WNT and WPHA. To take full advantage of the potential of AOPs for practical applications, such as ED identification and assessment, the need for more complex AOP networks (AOPNs) of inter-connected AOPs is recognized (Knapen et al., 2018). AOPNs provide further insight into different pathways that are connected in some way, either by contributing to the same AO or by sharing common KEs. This methodology also allows for identifying core KEs and KERs, i.e., events that are central to the development of a specific AO and/or shared by many AOPs. Core KE(R)s may be especially interesting to identify in a MoA analysis, target for testing or use as basis for development of new test methods. AOPNs can also support hazard and risk assessment of chemical mixtures, by providing frameworks for assessing the combined effects of chemicals affecting the same target tissue via different mechanisms (Villeneuve et al., 2018; Beronius et al., 2020).

For AOPs to have practical application, they should be described in a harmonized way and be publicly available. The AOP wiki (https://aopwiki.org/) provides such a repository of AOPs, and several AOPs describing perturbations to hormonal pathways leading to adverse effects are already included in the wiki (Audouze et al., 2021; Ravichandran et al., 2022; Wiklund et al., 2023). However, there is a need for further refinement of existing AOPs to improve overall confidence, as well as to develop additional AOPs describing relevant MoAs that are still missing. This includes reproductive toxicity where effects of EDs on both male and female reproductive systems have been well documented (Schwartz et al., 2019; 2021; Duursen van et al., 2020; Sharma et al., 2020; Rodprasert et al., 2021; You and Song, 2021; Delbes et al., 2022; Marlatt et al., 2022), yet where we still lack fundamental knowledge allowing us to predict with high confidence *in vivo* outcomes based on data from alternative test methods (Svingen et al., 2022).

The aim of this study was to conduct a systematic inventory of the AOP wiki to identify currently available AOPs that are relevant for mammalian reproductive toxicity and human health. The work was limited to AOPs relevant for EAS modalities. The study also set out to identify core KEs and KERs, i.e., KE(R)s shared by several of the identified AOPs and provide a basis for further AOP development to close gaps and support regulatory identification and assessment of EDs.

## 2 Materials and methods

**FIGURE 1:**
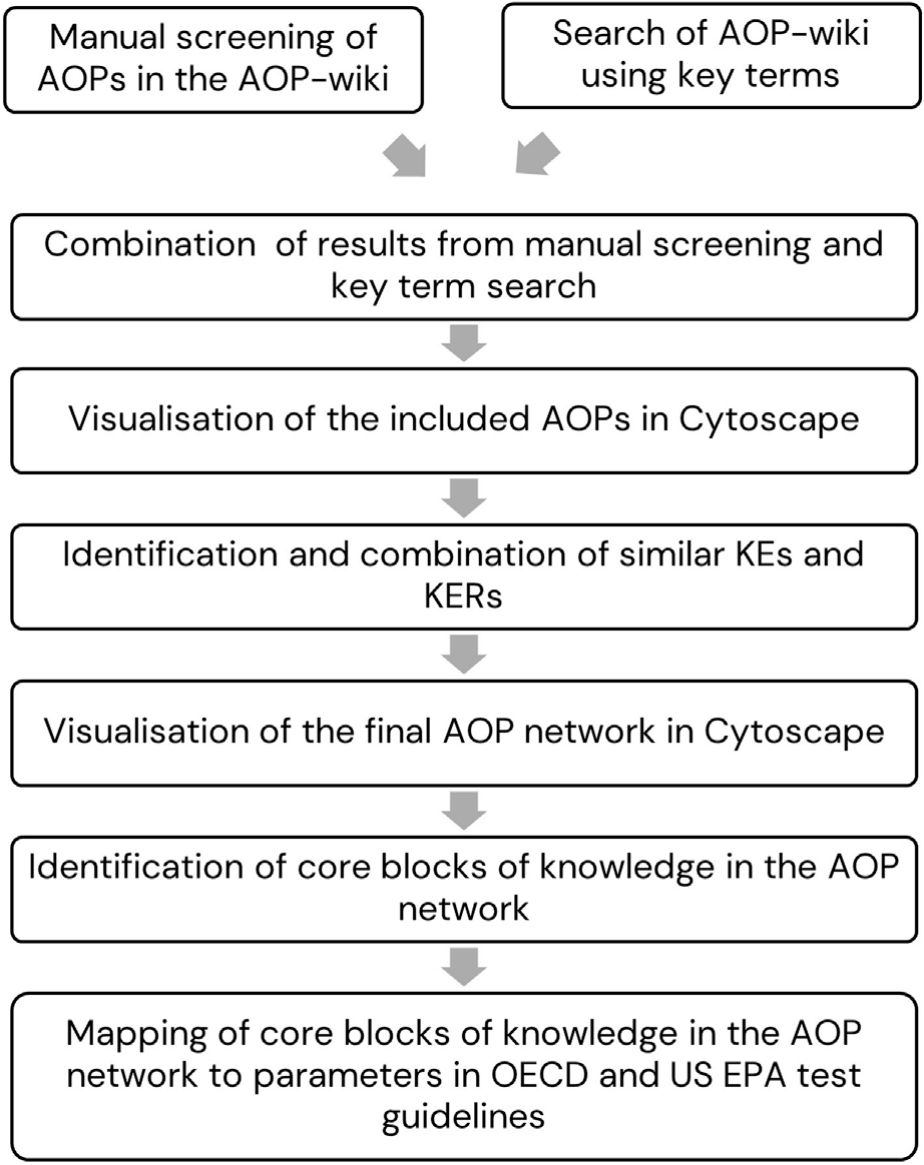
Outline of the methodology.

We applied an inclusive approach to capture all AOPs in the AOP wiki with relevance for EAS-mediated reproductive toxicity in mammals. The applied methodology was based on that developed and described by Wiklund et al. (2023), as outlined in Figure 1.

### 2.1 Development of key terms for identification of relevant AOPs

Key terms were derived from the following parameters listed in the ECHA/EFSA Guidance for the identification of EDs in the context of Regulations (EU) No 528/2012 and (EC) No 1107/2009 (ECHA/EFSA/JRC, 2018):

• *in vitro* mechanistic parameters
• *in vivo* mechanistic parameters for EAS modalities for mammals, fish, amphibians, birds
• EAS-mediated parameters for mammals, fish, amphibians, birds
• parameters sensitive to, but not diagnostic of, EATS for mammals, fish, amphibians, birds

Parameters for non-mammals (fish, amphibians, and birds) were included as key terms since AOPs described for non-mammal vertebrates may include early KEs relevant for mammals. Key terms are listed in Supplementary Material.

### 2.2 Manual screening of AOPs in the AOP wiki

The AOP wiki was screened to identify all AOPs relevant for mammalian reproductive toxicity mediated by EAS modalities. All AOPs in the AOP wiki were manually screened by reviewing each AOP, including reviewing the description of the KEs in the AOP, and cross-checking against the key terms. The screening was carried out independently by two reviewers in parallel on November 8, November 9, December 8 and 13 December 2022. Disagreements were resolved by discussion.

Relevant AOPs were identified based on the following inclusion and exclusion criteria using expert judgement:

Inclusion criteria:

• the AOP is relevant for reproductive toxicity mediated by EAS modalities, and
• the AOP describes effects related to identified key terms, and
• the AOP is relevant for mammalian or non-mammalian vertebrates, and
• the AOP is included in the AOP wiki with or without description of KEs or KER.

Exclusion criterion:

• The AOP was only relevant for invertebrate species.

### 2.3 Search of AOP wiki using key terms

To ensure that no relevant AOPs were overlooked in the manual screening, a search using the key terms listed in Supplementary Material was conducted in the AOP wiki as a subsequent step. This was considered appropriate due to potential errors and mismatches in information entered into the AOP wiki. The steps of the search are outlined in Figure 4. The searches were conducted by one reviewer on December 18–21, 2022.

First, the full texts of the AOP pages were searched by one key term at a time. The identified AOPs were listed. In the next step, the KE pages were searched by one key term at a time. The identified KEs were listed, and the relevance was assessed using the following criteria using expert judgement:

Inclusion criteria:

• the AOP, KE or KER is relevant for reproductive toxicity mediated by EAS modalities, and
• the AOP, KE or KER describes effects related to identified key terms, and
• the AOP, KE or KER is relevant for mammalian or non-mammalian vertebrates, and
• the AOP is included in the AOP wiki with or without description of KEs or KER.

Exclusion criteria:

• The key term was mentioned in the full text of an AOP-, KE- or KER-page but the AOP, KE or KER was clearly not relevant for reproductive toxicity mediated by EAS modalities, or
• The AOP was only relevant for invertebrate species.

The relevance was recorded. Thereafter AOPs that included the relevant KEs were identified and listed. Next, KER pages were searched by one key term at time. The identified KERs were listed and the relevance of the KERs was assessed using the same criteria as previously. Thereafter AOPs that included the relevant KERs were identified and listed. The lists of AOPs from the searches of the AOP, KE and KER pages were combined into one list and the relevance of the AOPs were assessed using the same criteria as previously.

### 2.4 Combination of results from manual screening and key term search

The lists of identified AOPs from the manual screening and the key term search were compared and combined. An additional check of the AOP wiki was done on 21 December 2022, to identify any relevant AOPs added after the screening and search. The AOP list was cross-checked by two additional reviewers and disagreements resolved before it was finalised.

### 2.5 Visualisation of the included AOPs in Cytoscape

The identified AOPs and their connections to each other were visualized using the Cytoscape software. Three tab-delimited files containing basic information relating to all KEs, KERs and KE components were downloaded from the AOP wiki on 23 January 2023. The IDs for the included AOPs were recorded in a separate file. The four files were imported into R-studio (https://www.rstudio.com/) and combined using an R script to produce one file with information on the included AOPs in a format suitable for import into Cytoscape (https://cytoscape.org) (Wiklund et al., 2023).

The file with the AOPs was imported into Cytoscape to visualise the included AOPs (Supplementary Material).

### 2.6 Identification and combination of similar KEs and KERs

**FIGURE 2:**
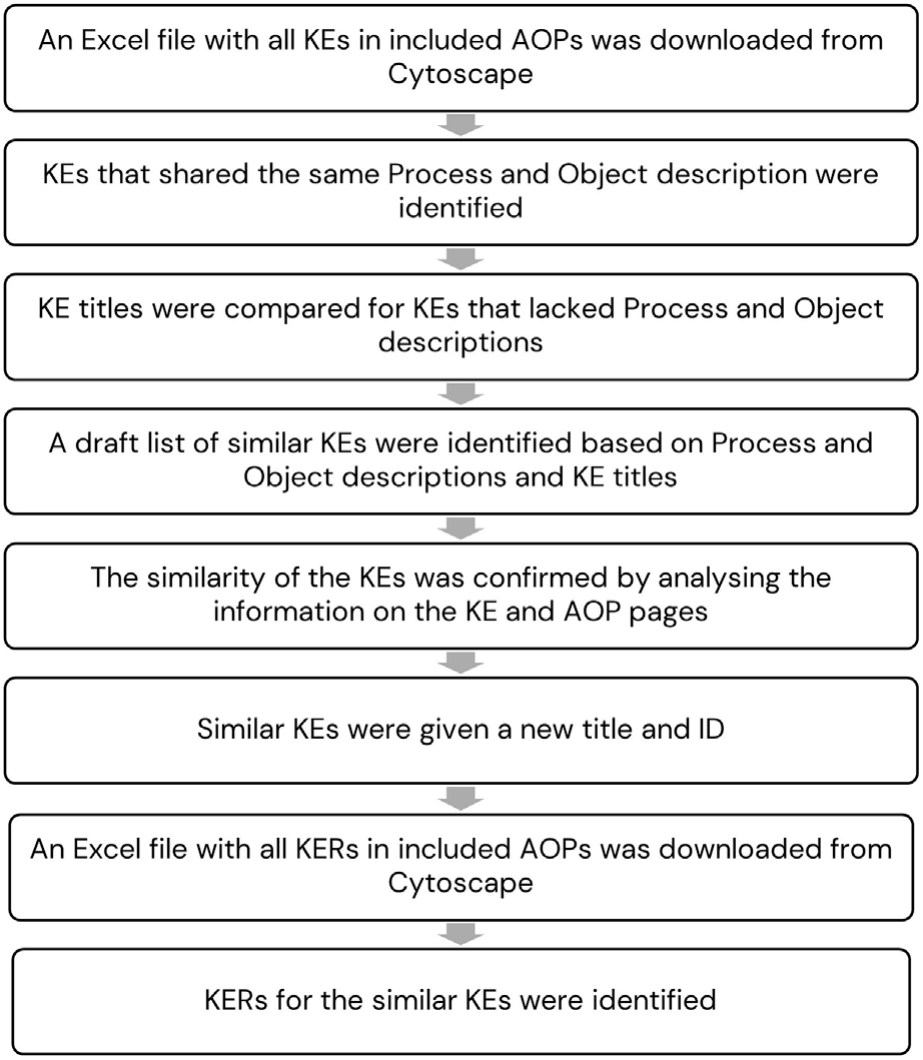
Steps in the methodology to identify similar KEs and KERs.

The AOP wiki contains many KEs that are the same, or very similar, but have been entered into the AOP wiki as separate KEs by different AOP developers. These KEs must be identified and combined in the input file for Cytoscape to create the final AOPN in Cytoscape, and to facilitate analyses of links between AOPs, and of core blocks of knowledge. The similar KEs were identified based on information in the AOP wiki and expert judgement. The steps in the methodology are outlined in Figure 2 and described below.

An Excel file with all KEs in the included AOPs in Cytoscape was downloaded from Cytoscape. The Excel file with the KEs included information from the AOP wiki on the Key event components Process and Object for each KE if available in the KE page in the AOP wiki. The Process describes the underlying biological system (e.g., receptor signalling). The Object is the subject of the perturbation (e.g., a specific biological receptor that is activated or inhibited). KEs that shared the same Process and Object description were first identified. Thereafter KE titles were compared for KEs that lacked Process and Object descriptions. Based on this a draft list of similar KEs were identified. The similarity of the KEs was confirmed by analysing the information on the KE pages and the AOP pages that included the KEs.

Similar KEs were combined and given a new KE title based on the title of the original KEs and adjusted according to the guidance for KE titles in the AOP Developers’ handbook (OECD, 2023). The KE ID of the combined KE included the IDs for all original KEs. A combined KE was labelled as MIE or AO based on the label of the original KEs. In cases where at least one of the original KEs was MIE or AO, the combined KE received that label.

An Excel file including all KERs in the included AOPs was downloaded from Cytoscape to identify the similar KERs. The similar KEs were first identified in the Excel file with KERs and thereafter could the corresponding similar KERs be identified.

The similar KEs and KERs were combined for the purpose of this study in Excel files and Cytoscape network but the information in AOP wiki was not changed.

### 2.7 Visualisation of the final AOPN in cytoscape

The file prepared using the R script that formed the input data for Cytoscape was manually modified by combining the similar KEs. The input file with the combined KEs was imported into Cytoscape to visualise the final AOPN (Supplementary Material).

### 2.8 Identification of core blocks of knowledge in the AOPN

Core blocks of knowledge are defined as KEs or KERs that are shared across various identified AOPs. The following criteria were used to identify core KEs and core KERs:

Criteria for core KEs:

• The KE is included in three or more AOPs, or the KE is included in two AOPs, is connected by three or more KERs and describes a central biological process as assessed by expert knowledge of the biological processes involved and the information in the AOP wiki, and
• The KE has a mammalian taxonomical applicability domain

Criteria for core KERs:

• The KER is included in three or more AOPs, and
• The KER has a mammalian taxonomical applicability domain

The following methodology was used to identify the core KEs and KERs. The input file for Cytoscape with the combined KEs was used to identify how many AOPs contained each of the KEs. The number of AOPs connected to each KE was recorded in an Excel file. The number of KERs connected to each KE was identified using the network analysis function in Cytoscape that calculated the degree of a node (KE) which is the number of edges (KERs) linked to the node. See also Villeneuve et al., 2018 for a discussion of network analytics. The number of connected KERs was recorded in the same Excel file as the number of AOPs. The taxonomical applicability domain described in the AOP wiki for the KEs was also noted in the Excel file. KEs that were included in two AOPs and connected by three or more KERs were analysed and based on expert knowledge of the biological processes involved and the information in the AOP wiki it was decided whether the KE describes a central biological process and should be considered a core KE. The identified core KEs based on the criteria and the described methodology were noted in the Excel file (Supplementary Material).

The input file for Cytoscape with the combined KEs was used to identify how many AOPs contained each of the KERs. The number of AOPs connected to each KER was recorded in an Excel file. The taxonomical applicability domain described in the AOP wiki for the KERs was also noted in the Excel file. The identified core KERs based on the criteria and the described methodology were noted in the Excel file (Supplementary Material).

### 2.9 Mapping of core blocks of knowledge in the AOPN to parameters in OECD and US EPA test guidelines

To identify whether the core blocks of knowledge in the AOPN are included in the current tests for identification of endocrine disruptors, the identified core KEs were mapped against parameters in OECD and US EPA test guidelines described in ECHA/EFSA/JRC (2018).

## 3 Results

### 3.1 Manual screening of AOPs in the AOP wiki

A total of 56 AOPs were included based on the manual screening of the AOP wiki for AOPs relevant for mammalian reproductive toxicity mediated by EAS modalities (Figure 4).

### 3.2 Search of AOP wiki using key terms

**FIGURE 3:**
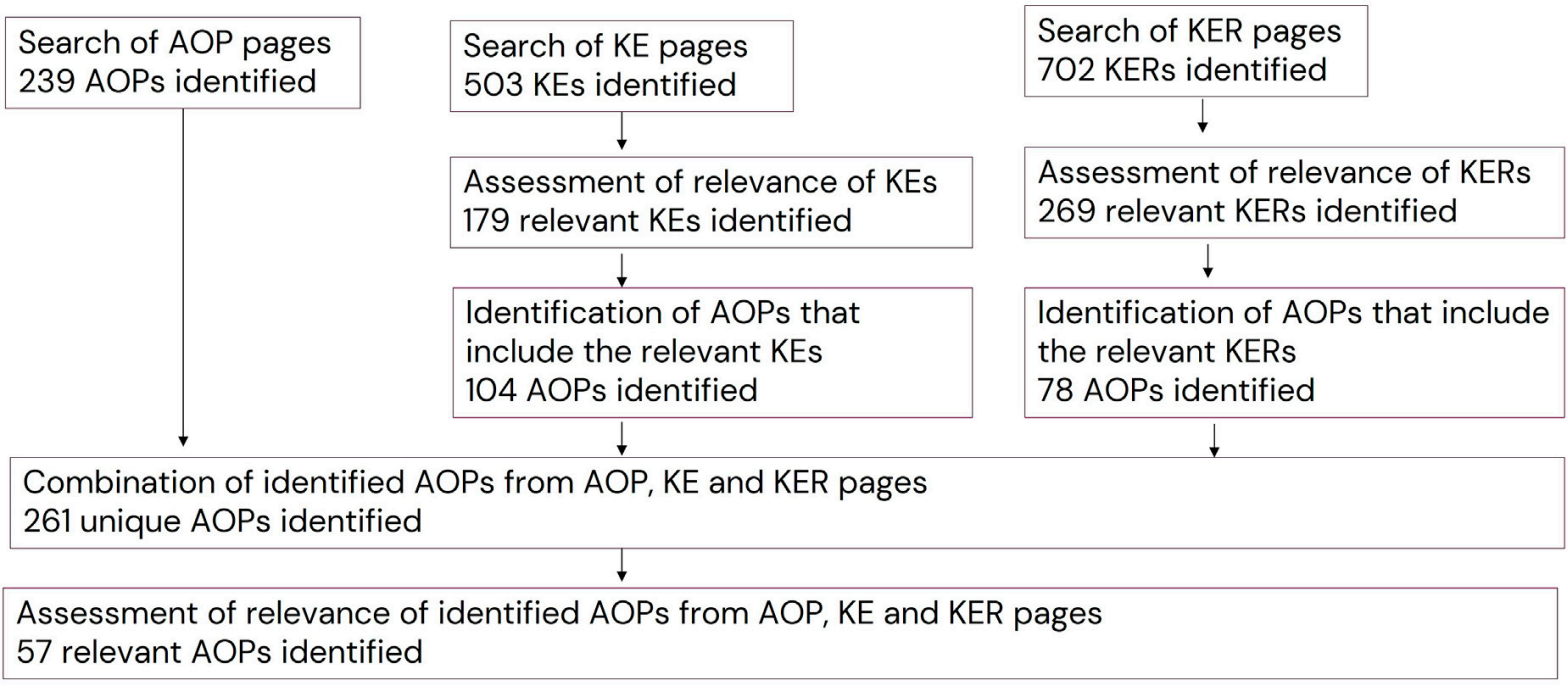
Results for search of AOP wiki using key terms.

The key term search of the AOP and KE pages identified 239 AOPs and 503 individual KEs, respectively. Out of the identified KEs, 179 were considered relevant for mammalian reproductive toxicity. The relevant KEs were included in 104 AOPs. In addition, 702 KERs were identified in the search of the KER pages, and 269 were considered relevant. The relevant KERs were included in 78 AOPs. By combining the lists of AOPs from the searches of the AOP, KE and KER pages, 261 unique AOPs were identified. Out of these, 57 were considered relevant for inclusion in an AOPN describing EAS-mediated reproductive toxicity (Figure 3).

### 3.3 Combining results from manual screening and search using key terms

**FIGURE 4:**
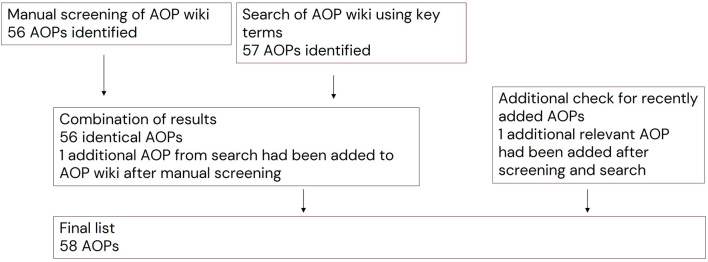
Combining results from manual screening and search using key terms.

The list of AOPs identified by manual screening was compared to the search using key terms. The lists were identical except for one additional AOP (ID 476), which had been added to the AOP wiki after the manual screening and before the key term search. Checking of recently added AOPs identified one additional relevant AOP that had been added to the AOP wiki after the key term search (ID 477). This resulted in inclusion of in total 58 AOPs (Figure 4).

The final list of 58 AOPs is outlined in Table 1 and in Supplementary Material. Importantly, no assessment of completeness or reliability of the included AOPs was performed since the aim was to identify all relevant AOPs as a starting point for further analysis and development. As shown in Supplementary Material, only four of the AOPs have been endorsed by WPHA/WNT and three are under review.

**TABLE 1:** Fifty-eight AOPs were identified as relevant from the AOP wiki.

AOP ID	AOP title	Included in final AOPN after combination of KEs
7	Aromatase (Cyp19a1) reduction leading to impaired fertility in adult female	Yes
18	PPARα activation in utero leading to impaired fertility in males	Yes
19	Androgen receptor antagonism leading to adverse effects in the male foetus (mammals)	Yes
**23**	**Androgen receptor agonism leading to reproductive dysfunction (in repeat-spawning fish)**	Yes
**25**	**Aromatase inhibition leading to reproductive dysfunction**	Yes
29	Estrogen receptor agonism leading to reproductive dysfunction	Yes
30	Estrogen receptor antagonism leading to reproductive dysfunction	Yes
51	PPARα activation leading to impaired fertility in adult male rodents	Yes
52	ER agonism leading to skewed sex ratios due to altered sexual differentiation in males	No
64	Glucocorticoid Receptor (GR) Mediated Adult Leydig Cell Dysfunction Leading to Decreased Male Fertility	Yes
66	Modulation of Adult Leydig Cell Function Subsequent Glucocorticoid Activation in the Fetal Testis	No
67	Modulation of Adult Leydig Cell Function Subsequent to Estradiaol Activation in the Fetal Testis	No
68	Modulation of Adult Leydig Cell Function Subsequent to Alterations in the Fetal Testis Protome	No
69	Modulation of Adult Leydig Cell Function Subsequent to Decreased Cholesterol Synthesis or Transport in the Adult Leydig Cell	No
70	Modulation of Adult Leydig Cell Function Subsequent to Proteomic Alterations in the Adult Leydig Cell	No
71	Modulation of Adult Leydig Cell Function Subsequent to Glucocorticoid Activation	No
73	Xenobiotic Inhibition of Dopamine-beta-Hydroxylase and subsequent reduced fecundity	No
74	Modulation of Adult Leydig Cell Function Subsequent to Hypermethylation in the Fetal Testis	No
102	Cyclooxygenase inhibition leading to reproductive dysfunction via interference with meiotic prophase I/metaphase I transition	Yes
103	Cyclooxygenase inhibition leading to reproductive dysfunction via interference with spindle assembly checkpoint	Yes
111	Decrease in androgen receptor activity leading to Leydig cell tumors (in rat)	Yes
112	Increased dopaminergic activity leading to endometrial adenocarcinomas (in Wistar rat)	Yes
120	Inhibition of 5α-reductase leading to Leydig cell tumors (in rat)	Yes
122	Prolyl hydroxylase inhibition leading to reproductive dysfunction via increased HIF1 heterodimer formation	Yes
123	Unknown MIE leading to reproductive dysfunction via increased HIF-1alpha transcription	Yes
124	HMG-CoA reductase inhibition leading to decreased fertility	Yes
126	Alpha-noradrenergic antagonism leads to reduced fecundity via delayed ovulation	No
146	Estrogen Receptor Activation and Female Precocious Puberty	No
153	Aromatase Inhibition leading to Ovulation Inhibition and Decreased Fertility in Female Rats	No
165	Antiestrogen activity leading to ovarian adenomas and granular cell tumors in the mouse	Yes
167	Early-life estrogen receptor activity leading to endometrial carcinoma in the mouse.	Yes
168	GnRH pulse disruption leading to mammary adenomas and carcinomas in the SD rat.	Yes
169	GnRH pulse disruption leading to pituitary adenomas and carcinomas in the SD rat.	Yes
199	ER mediated breast cancer AOP	No
200	Estrogen receptor activation leading to breast cancer	Yes
271	Inhibition of thyroid peroxidase leading to impaired fertility in fish	Yes
288	Inhibition of 17α-hydrolase/C 10,20-lyase (Cyp17A1) activity leads to birth reproductive defects (cryptorchidism) in male (mammals)	Yes
289	Inhibition of 5α-reductase leading to impaired fecundity in female fish	Yes
295	Early-life stromal estrogen receptor activation by endocrine disrupting chemicals in the mammary gland leading to enhanced cancer risk	No
305	5α-reductase inhibition leading to short anogenital distance (AGD) in male (mammalian) offspring	Yes
306	Androgen receptor (AR) antagonism leading to short anogenital distance (AGD) in male (mammalian) offspring	Yes
307	Decreased testosterone synthesis leading to short anogenital distance (AGD) in male (mammalian) offspring	Yes
309	Luteinizing hormone receptor antagonism leading to reproductive dysfunction	Yes
310	Embryonic Activation of the AHR leading to Reproductive failure, via epigenetic down-regulation of GnRHR	Yes
323	PPARalpha Agonism Impairs Fish Reproduction	Yes
344	Androgen receptor (AR) antagonism leading to nipple retention (NR) in male (mammalian) offspring	Yes
345	Androgen receptor (AR) antagonism leading to decreased fertility in females	Yes
**346**	**Aromatase inhibition leads to male-biased sex ratio via impacts on gonad differentiation**	Yes
348	Inhibition of 11β-Hydroxysteroid Dehydrogenase leading to decreased population trajectory	Yes
349	Inhibition of 11β-hydroxylase leading to decresed population trajectory	Yes
372	Androgen receptor antagonism leading to testicular cancer	No
**376**	**Androgen receptor agonism leading to male-biased sex ratio**	Yes
440	Hypothalamic estrogen receptors inhibition leading to ovarian cancer	Yes
443	Alcohol Induced DNA damage and mutations leading to Metastatic Breast Cancer	Yes
445	Estrogen Receptor Alpha Agonism leads to Impaired Reproduction	Yes
465	Alcohol dehydrogenase leading to reproductive dysfunction	Yes
476	Adverse Outcome Pathways diagram related to PBDEs associated male reproductive toxicity	No
477	Androgen receptor (AR) antagonism leading to hypospadias in male offspring	Yes

Out of these, 13 did not include any KERs and 3 included neither KEs nor KERs and were excluded from the final AOPN (shaded rows). Four of the AOPs, ID numbers 23, 25, 346, and 376, have so far been endorsed by the WPHA/WNT and are indicated by bold text.

### 3.4 Initial visualisation of the included AOPs in cytoscape

The 58 included AOPs and their connections to each other were visualised using Cytoscape. Manual analysis of the AOPs in Cytoscape showed 5 networks and 3 single AOPs not connected to any of the networks. Thirteen AOPs did not include any KERs and 3 AOPs included neither KEs nor KERs (Supplementary Material).

### 3.5 Identification and combination of similar KEs and KERs

Similar KEs in the included AOPs were identified based on information in the AOP wiki and expert judgement. Sixty-eight KEs were identified to be similar to a different KE and were combined in order to create the final AOPN (Table 2 and Supplementary Material). Thirty-five KERs were identified to be similar to a different KER (Table 3 and Supplementary Material).

**TABLE 2:** Similar KEs that were combined to create the final AOPN.

Title of combined KEs	IDs of combined KEs
Altered, Transcription of genes by androgen receptor	286, 1687
Decrease population	360, 679
Decreased, Androgen receptor activity	27, 742, 1614
Decreased, dihydrotestosterone	792, 1613
Decreased, Estrogen receptor activity	112, 1046
Decreased, gonadotropin releasing hormone	530, 689, 969, 1071
Decreased, gonadotropins	129, 1986
Decreased, luteinizing hormone	531, 690, 970, 1072
Decreased, testosterone	446, 808, 1612, 1690
Hyperplasia, Mammary gland	1078, 1192
Impaired Ovulation	532, 971, 1074, 1695
Impaired, fertility-reproduction	330, 337, 406, 527, 675, 1863, 1991
Impaired, Spermatogenesis	505, 520, 543, 646, 1758, 1798
Increased, Breast Cancer	1079, 1193
Increased, Endometrial adenocarcinomas	773, 1070
Increased, Estrogen receptor activity	111, 748, 1065, 1181
Increased, hyperplasia glandular epithelial cells of endometrium	772, 1069
Increased, hyperplasia Leydig cell	415, 744
Increased, luteinizing hormone	414, 754, 791, 1050
Inhibition, 5-alpha-reductase	790, 1617
Inhibition, Aromatase	36, 408, 964
Malformation, Male reproductive tract	240, 348, 809

**TABLE 3:** Similar KERs in the final AOPN.

Titles of similar KERs	IDs of similar KERs
a) Altered, Transcription of genes by AR leads to AGD, decreased	2127, 2129
b) Decrease, transcription of genes by AR leads to AGD, decreased	
a) Decrease, testosterone level leads to Decrease, AR activation	1936, 2131
b) Reduction, testosterone levels leads to Decrease, AR activation	
a) Decrease, testosterone level leads to Decrease, DHT level	1934, 2126
b) Reduction, testosterone levels leads to Decrease, DHT level	
a) Decreased sperm quantity or quality in the adult, Decreased fertility leads to impaired, Fertility	1650, 2161, 2274
b) Decreased spermatogenesis leads to impaired, Fertility	
c) Impaired, Spermatogenesis leads to impaired, Fertility	
a) Decreased, 11KT leads to Impaired, Spermatogenesis	2076, 2166
b) Decreased, 11KT leads to Decreased spermatogenesis	
a) Feminisation or incomplete development, Primary and accessory male sex organs leads to N/A, Impairment of reproductive capacity	266, 405, 808
b) Malformation, Male reproductive tract leads to impaired, Fertility	
c) Malformed, Male reproductive tract leads to Decrease, Fertility	
a) Hyperplasia, Mammary gland leads to Increased, Adenomas/carcinomas (mammary)	1116, 1252
b) Hyperplasia, Mammary gland leads to Increased, Adenomas/carcinomas (mammary)	
a) Increase, Hyperplasia (glandular epithelial cells of endometrium) leads to Increase, Endometrial adenocarcinomas	771, 1107
b) Increased, Hyperplasia (glandular epithelial cells of endometrium) leads to Increased, adenosquamous carcinomas of endometrium	
a) Increased, Luteinizing hormone (LH) leads to Increase, Hyperplasia (Leydig cells)	745, 794
b) Increased, Leutinizing hormone (LH) leads to Increase, Hyperplasia (Leydig cells)	
a) Inhibition, 5α-reductase activity leads to Decrease, Bioactivation of testosterone	792, 1880
b) 5α-reductase, inhibition leads to Decrease, DHT level	
a) Inhibition, Aromatase leads to Reduction, 17beta-estradiol synthesis by ovarian granulosa cells	45, 396
b) reduction in ovarian granulosa cells, Aromatase (Cyp19a1) leads to Reduction, 17beta-estradiol synthesis by ovarian granulosa cells	
a) N/A, Androgen receptor, Antagonism leads to Altered, Transcription of genes by AR	33, 2124, 2128
b) Decrease, AR activation leads to Altered, Transcription of genes by AR	
c) Decrease, AR activation leads to decrease, transcription of genes by AR	
a) Reduced, Gonadotropin releasing hormone, hypothalamus leads to Reduced, Luteinizing hormone (LH), plasma	691, 1108
b) Decreased, GnRH pulsatility/release in hypothalamus leads to Decreased, LH Surge from anterior pituitary	
a) Reduced, Reproductive Success leads to Decrease, Population growth rate	675, 2292
b) impaired, Fertility leads to Decrease, Population growth rate	
a) Reduction, testosterone level leads to Malformation, Male reproductive tract	608, 807
b) Decreased, Testosterone leads to malformed, Male reproductive tract	
a) Reduction, Testosterone synthesis in Leydig cells leads to Reduction, testosterone level	439, 2125
b) Reduction, Testosterone synthesis in Leydig cells leads to reduction, testosterone levels	

### 3.6 Visualisation of the final AOPN in cytoscape

**FIGURE 5:**
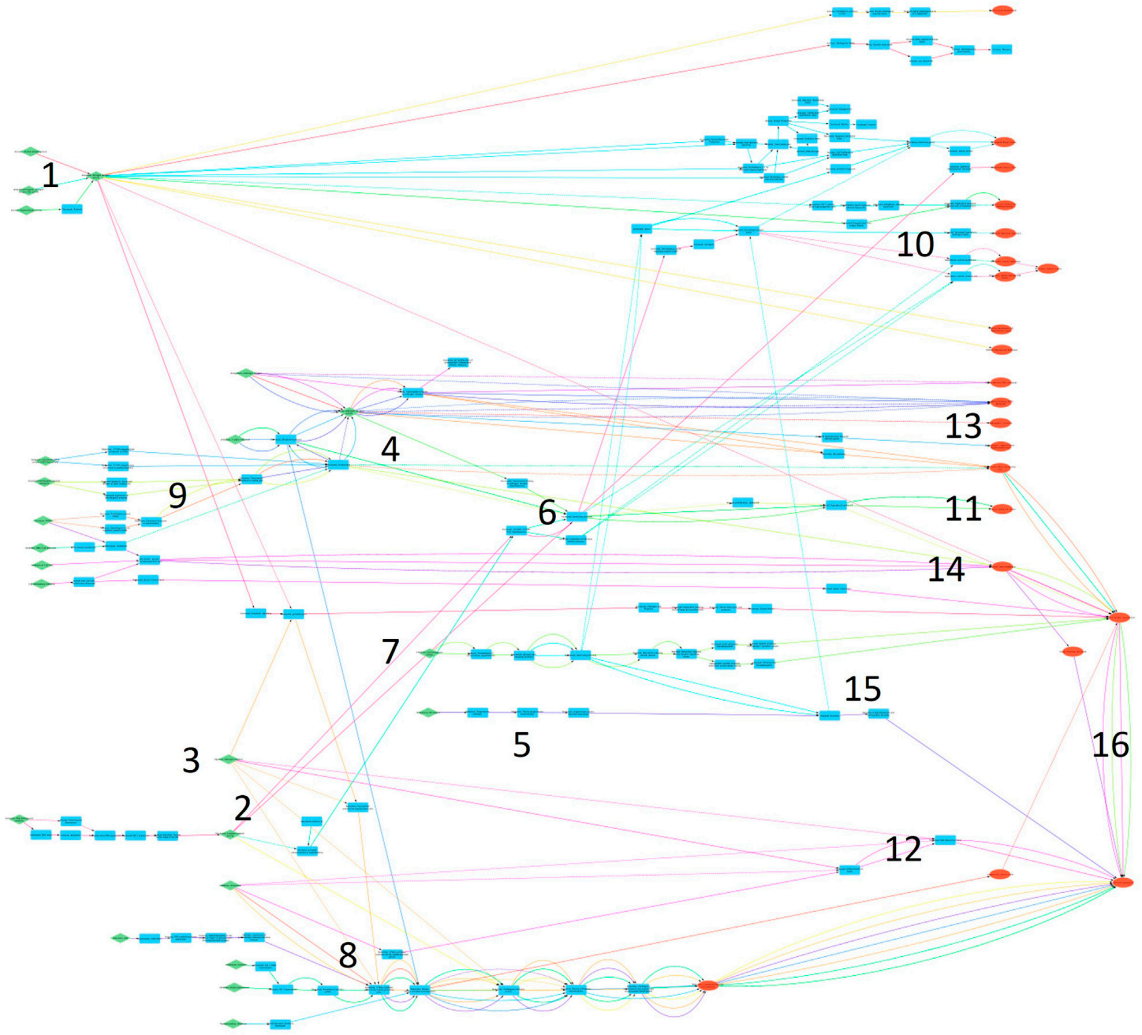
Overview of the final AOPN. MIEs are shown in green, AOs in red and other KEs in blue. Subnetworks are labelled with numbers, see Table 5.

The included AOPs before the combination of similar KEs included five networks and three single AOPs. Combination of the similar KEs connected the five networks and the three single AOPs resulted in a final large AOPN in Cytoscape that included all identified AOPs, except for the ones that did not have any KEs or KERs. Figure 5 gives on overview of the final AOPN (a high-resolution figure is provided in Supplementary Material). Table 4 summarises the AOPs, KEs and KERs in the final AOPN. The AOPs, KEs and KERs are listed in Supplementary Material.

**TABLE 4:** Summary of the final AOPN.

	Number
Total number of AOPs identified	58
Number of AOPs included in the network	42
Total number of KEs identified (including MIEs and AOs)	252
Number of KEs included in the network (including MIEs and AOs)	191
Number of KEs not included in the network (without KERs) (including MIEs and AOs)	61
Number of KERs identified and included in the network	299
Number of MIEs included in the network	23
Number of AOs included in the network	22

### 3.7 Subnetworks

Several subnetworks can be identified from the large AOPN (Table 5; Figure 5). Nine subnetworks describe effects on hormone levels or hormone activity, two subnetworks are related to cancer outcomes, three are related to the male reproductive system, one is related to the female reproductive system and one to overall effects on fertility and reproduction. The subnetworks include between 1-22 KEs and 1-20 AOPs each (Table 5). Specific KEs and AOPs in each subnetwork is in Supplementary information.

**TABLE 5:** Subnetworks identified including the number of KEs and AOPs included in each subnetwork.

	Number of included KEs	Number of included AOPs
Hormones		
1. Increased ER activity	5	6
2. Decreased ER activity	1	4
3. AR agonism	1	2
4. AR antagonism and decreased AR activity	4	9
5. Progesterone	4	1
6. HPG axis, increased GnRH, LH, FSH	3	5
7. HPG axis, decreased GnRH, FSH, LH	6	6
8. Steroidogenesis, estrogens	19	10
9. Steroidogenesis, androgens	22	12
Cancer		
10. Breast, endometrial, ovarian and pituitary cancer	38	8
11. Leydig cell tumours	3	3
Male reproductive system		
12. Testis differentiation and sex ratio	2	2
13. NR, AGD, hypospadias, cryptorchidism, malformation male reproductive tract	7	9
14. Impaired spermatogenesis	1	5
Female reproductive system		
15. Oocyte maturation, ovulation, ovarian cycle	5	6
Overall effects on fertility and reproduction		
16. Fertility, reproduction and population decrease	3	20

### 3.8 Identification of core blocks of knowledge in the AOPN

Overall, 26 KEs and 11 KERs were identified as core blocks of knowledge in the AOPN (Tables 6, 7; Supplementary Material). Only KEs and KERs relevant for mammalian reproductive toxicity were considered for identification of core blocks of knowledge.

**TABLE 6:** Core KEs in the AOPN.

KE ID	KE type	KE title	Number of KERs connected to the KE	Number of AOPs that include the KE
360-679	AO	Decrease population	15	16
330-337-406-527-675-1863-1991	AO	Impaired, fertility-reproduction	17	11
219	KE	Reduction, Plasma 17beta-estradiol concentrations	19	8
27-742-1614	MIE	Decreased, Androgen receptor activity	19	8
111-748-1065-1181	MIE	Increased, Estrogen receptor activity	16	6
446-808-1612-1690	KE	Decreased, testosterone	15	6
3	KE	Reduction, 17beta-estradiol synthesis by ovarian granulosa cells	13	6
286-1687	KE	Altered, Transcription of genes by androgen receptor	11	6
505-520-543-646-1758-1798	AO	Impaired, Spermatogenesis	8	6
792-1613	KE	Decreased, dihydrotestosterone	10	5
414-754-791-1050	KE	Increased, luteinizing hormone	10	5
26	MIE	Antagonism, Androgen receptor	6	5
531-690-970-1072	KE	Decreased, luteinizing hormone	10	4
413	KE	Reduction, Testosterone synthesis in Leydig cells	8	4
1688	AO	anogenital distance (AGD), decreased	6	4
530-689-969-1071	KE	Decreased, gonadotropin releasing hormone	6	4
112-1046	MIE	Decreased, Estrogen receptor activity	5	4
1076	KE	Increased, circulating estrogen levels	8	3
240-348-809	AO	Malformation, Male reproductive tract	7	3
1756	KE	Decreased, plasma 11-ketotestosterone level	6	3
415-744	KE	Increased, hyperplasia Leydig cell	6	3
36-408-964	MIE	Inhibition, Aromatase	5	3
790-1617	MIE	Inhibition, 5-alpha-reductase	3	3
227	MIE	Activation, PPAR alpha	3	3
25	MIE	Agonism, Androgen receptor	6	2
532-971-1074-1695	KE	Impaired Ovulation	5	2

**TABLE 7:** Core KERs in the AOPN.

KER ID	KER title	Number of AOPs that include the KER
5	Reduction, 17beta-estradiol synthesis by ovarian granulosa cells leads to Reduction, Plasma 17beta-estradiol concentrations	6
33-2124-2128	Decreased, Androgen receptor activity leads to Altered, Transcription of genes by androgen receptor	4
439-2125	Reduction, Testosterone synthesis in Leydig cells leads to Decreased, testosterone	4
675-2292	Impaired, fertility-reproduction leads to Decrease population	4
691-1108	Decreased, gonadotropin releasing hormone leads to Decreased, luteinizing hormone	4
1935	Decreased, dihydrotestosterone leads to Decreased, Androgen receptor activity	3
2130	Antagonism, Androgen receptor leads to Decreased, Androgen receptor activity	3
2820	Decreased, Androgen receptor activity leads to AGD, decreased	3
1650-2161-2274	Impaired, Spermatogenesis leads to Impaired, fertility-reproduction	3
266-405-808	Malformation, Male reproductive tract leads to Impaired, fertility-reproduction	3
792-1880	Inhibition, 5-alpha-reductase leads to Decreased, dihydrotestosterone	3

### 3.9 Mapping of core blocks of knowledge in the AOPN to parameters in OECD and US EPA test guidelines

Out of the 26 KEs identified as core blocks of knowledge in the AOPN, 19 were found to be included as parameters in current OECD and US EPA test guidelines (Table 8).

**TABLE 8:** Core KEs included in current OECD Guidelines for the testing of chemicals and US EPA test guidelines.

KE ID	KE type	KE title	Parameter(s) measured	Test guideline (OECD, US EPA)
227	MIE	Activation, PPAR alpha	*none*	*none*
25	MIE	Agonism, Androgen receptor	Androgen receptor binding/transactivation	890.1150; 458
286-1687	KE	Altered, Transcription of genes by androgen receptor	*none*	*none*
1688	AO	anogenital distance (AGD), decreased	Anogenital distance	414; 421; 422; 426; 416; 443
26	MIE	Antagonism, Androgen receptor	Androgen receptor binding/transactivation	890.1150; 458
360-679	AO	Decrease population	*none*	*none*
1756	KE	Decreased, plasma 11-ketotestosterone level	*none*	*none*
27-742-1614	MIE	Decreased, Androgen receptor activity	Androgen receptor binding/transactivation	890.1150; 458
792-1613	KE	Decreased, dihydrotestosterone	*none*	*none*
112-1046	MIE	Decreased, Estrogen receptor activity	Estrogen receptor binding/transactivation	455; 493; 890.1250
530-689-969-1071	KE	Decreased, gonadotropin releasing hormone	*none*	*none*
531-690-970-1072	KE	Decreased, luteinizing hormone	Luteinising hormone (LH) level	441; 408
446-808-1612-1690	KE	Decreased, testosterone	Testosterone level	441; 408
532-971-1074-1695	KE	Impaired Ovulation	Oestrus cyclicity	407; 408; 421; 422; 416; 443
330-337-406-527-675-1863-1991	AO	Impaired, fertility-reproduction	Fertility; Reproduction	415; 421; 422; 416; 443
505-520-543-646-1758-1798	AO	Impaired, Spermatogenesis	Sperm morphology; Sperm motility; Sperm numbers	408; 416; 443
1076	KE	Increased, circulating estrogen levels	Oestradiol level	441; 408
111-748-1065-1181	MIE	Increased, Estrogen receptor activity	Estrogen receptor binding/transactivation	455; 493; 890.1250
415-744	KE	Increased, hyperplasia Leydig cell	Testis histopathology	407; 408; 415; 421; 422; 451-3; 416; 443; 890-1500
414-754-791-1050	KE	Increased, luteinizing hormone	Luteinising hormone (LH) level	441; 408
790-1617	MIE	Inhibition, 5-alpha-reductase	*none*	*none*
36-408-964	MIE	Inhibition, Aromatase	Aromatase	890.1200
240-348-809	AO	Malformation, Male reproductive tract	Genital abnormalities	414; 415; 421; 422; 416; 443
3	KE	Reduction, 17beta-estradiol synthesis by ovarian granulosa cells	Steroidogenesis (oestradiol and/or testosterone synthesis); Steroidogenesis (genes/enzyme changes)	456; 441
219	KE	Reduction, Plasma 17beta-estradiol concentrations	Oestradiol level	441; 408
413	KE	Reduction, Testosterone synthesis in Leydig cells	Steroidogenesis (oestradiol and/or testosterone synthesis); Steroidogenesis (genes/enzyme changes)	456; 441

Shaded rows indicate core KEs currently not covered in standardized test guidelines.

## 4 Discussion

This paper describes an AOPN for mammalian reproductive toxicity mediated by the EAS modalities. The construction of the network was based on a systematic inventory of the AOP wiki, where a total of 58 single AOPs were identified as relevant and 42 of these contained sufficient information to be connected into one large network. The network includes 23 MIEs, 22 AOs and 145 other KEs, demonstrating the complexity and interconnectivity of mammalian reproductive toxicity mediated by endocrine mechanisms. The AOPN provides a basis for different applications, such as the identification of needs for future development of KEs, AOs or KERs that are relevant for ED assessment but are currently missing. To this end, we made an initial organisation of the AOPN and identified several subnetworks that describe effects on different hormone pathways and on specific AOs, including effects on male and female reproduction and endocrine-related cancers. Additional filters can be added to the AOPN to refine it further for different applications, for example, to highlight areas of the network relevant for specific taxa or life stages (Knapen et al., 2018), or focus on AOs that are of specific relevance for a certain assessment.

We specifically used the AOPN to identify core KEs and KERs that are common for several existing AOPs and that may be considered central events in EAS-mediated reproductive toxicity. Out of the 26 identified core KEs, 19 are already included as parameters measured in current OECD and US EPA test guidelines, indicating that they are relevant for regulatory ED testing and assessment. Further analyses of the core events for which standard tests are lacking are needed to ascertain their relevance, as well as the practicality and feasibility of measuring them, before any recommendations can be made regarding development and standardization of new tests. Additional potential applications of the AOPN include use as a framework for investigating connections between different pathways within the network that can influence effects in target organs, as well as for assessing combined effects of mixtures containing EDs.

A rigorous multi-step process was developed to identify relevant AOPs for inclusion in the network. This methodology should be useful for creating other AOPNs. It includes searches of the AOP wiki by two different methods, i.e., manually screening each AOP in the wiki by two reviewers and searching the wiki pages using pre-defined search terms, carried out by one reviewer. The results were then cross-checked by a third party and discussed until consensus was reached. Both methods identified the same AOPs in this case and it may seem unnecessarily complex to do both. However, exploring both methods is merited from a wider perspective. Manual review of each AOP in the wiki by an expert can reduce the risk of missing potentially relevant AOPs, as well as prevent the inclusion of irrelevant or unsuitable AOPs. The current AOP wiki is small enough to allow for a manual screening process. As more AOPs are added in the future, however, an efficient search methodology using key terms will be necessary. One limitation in searching the AOP wiki using key terms is that the present format of the wiki does not allow for applying strategies to search for several key terms or phrases simultaneously. Instead, the separate AOP, KE and KER pages must be searched individually using all search terms; a time-consuming process that also increases the risk for potential errors. Future developments to the AOP wiki should enable efficient search strategies to make more efficient use of all the available information (Wiklund et al., 2023).

The identification of AOPs for inclusion in the network relied to a significant extent on expert judgment regarding the relevance of specific AOPs (or parts of AOPs) for describing EAS-mediated reproductive toxicity. For example, the screening of the AOP wiki initially resulted in several differences in the AOPs identified as relevant by the two reviewers, which had to be resolved by discussions. It is therefore likely that AOPNs created by different people will identify slightly different AOPNs, even if developed for the same toxicity outcome(s). This underscores the importance of applying a structured approach and transparent reporting.

To visualize the AOPN, we used a computational workflow to automatically process, filter, and format the AOP data for use in Cytoscape (Wiklund et al., 2023). Visualization in Cytoscape allowed us to identify KEs that were similar or identical, as well as KE(R)s that were missing or incorrectly described in the wiki. The automated workflow facilitates updating and regenerating networks once such issues have been resolved, as it standardises and speeds up the process. Here, similar KEs were combined by reviewing the similarity between KE titles and descriptions, and application of expert judgment. This approach was considered appropriate for the purposes of this project, but it should be noted that whenever this practice is used, it needs to be carefully considered to be fit for purpose to ensure that no critical information from individual KE descriptions is lost. In this project the information in the AOP wiki for each combined KE will be analysed and used in further development of AOPs. Similarly, single KEs that were identified as relevant for this project, but were not part of an AOP, were excluded in the final AOPN but will be considered in during continued AOP(N) development.

New AOPs are continuously being added to the wiki and an additional manual screening of the AOP wiki was performed in August 2023 to identify any relevant AOPs that had been added after the initial AOP search. Four new relevant AOPs were identified: AOP 495 “Androgen receptor activation leading to prostate cancer,” AOP 496 “Androgen receptor agonism leading to reproduction dysfunction in zebrafish,” AOP 503 “Activation of uterine estrogen receptor-alfa leading to endometrial adenocarcinoma, via epigenetic modulation,” and AOP 504 “SULT1E1 inhibition leading to uterine adenocarcinoma via increased estrogen availability at target organ level.” All four AOPs include some KEs and AOs that were not previously described in the AOPN and will be considered when the AOPN is further developed.

Other AOPNs describing perturbations to different endocrine pathways have recently been described, albeit for different purposes and with different scopes. Several studies describe the development of AOPNs containing novel pathways as an alternative to postulating single AOPs, for example, related to disrupted female pubertal onset (Franssen et al., 2022), male reproductive tract abnormalities (Palermo et al., 2021), and for effects on male and female reproductive function in fish (Ankley et al., 2023; Morshead et al., 2023). Others describe AOPNs for specific types of endocrine mediated toxicities and stressors, such as exploring male reproductive toxicity of silver nanoparticles (Kose et al., 2023) and inorganic arsenic (Chai et al., 2021), or the impact of phthalates on female reproduction (Pogrmic-Majkic et al., 2022). In contrast, the AOPN developed here combines existing AOPs related to mammalian reproductive toxicity and provides a broad view of mechanisms and effects related to disruption of the EAS modalities for the purpose of further AOP development. Similar networks have been described by Wiklund et al. (2023) and Ravichandran et al. (2022), however these works focus primarily on development of methods for AOPN construction, filtering, and analyses.

There are limitations that need to be considered when using this and other AOPNs for further scientific or regulatory applications. Importantly, many AOPs in the AOP wiki are not yet fully developed, peer-reviewed, or finally endorsed by the OECD. This is also reflected in the AOPs included in this network; only four of the AOPs have been endorsed by WPHA/WNT and three are currently under review, although many are mature with respect to their development status. Consequently, there is no guarantee that all AOPs included in a network are of sufficient quality to justify using them as basis for conclusions on toxicological hazards. Any information in the AOP wiki, including the AOPN described herein, must therefore be carefully assessed for reliability before the information is used for scientific or regulatory purposes. Such assessment has not yet been conducted for the AOPN we have elaborated here, and it should thus only be viewed as a starting point for further analyses and AOP developments, activities that are currently being conducted.

Another limitation of using AOPNs is that the AOP wiki does not include AOPs for all mechanisms and AOs relevant for different toxicities. Any AOPN must therefore be assumed to be missing information necessary to, for example, reliably identify all relevant core KE(R)s. Indeed, we acknowledge that there are several putative or complete AOPs relevant for EAS-mediated reproductive toxicity published in the open literature but not necessarily entered into the wiki. For example, there are several AOPs describing effects on spermatogenesis and the development of the male reproductive tract, as well as nipple retention in rodents, or prostate and testis tumours caused by androgen receptor (AR) antagonism, estrogen receptor (ER) agonism or reduced testosterone levels via different mechanisms such as inhibition of aromatase, oxidative stress, AhR signalling, and retinoic acid signalling (Christiansen et al., 2020; Draskau et al., 2020; Gray et al., 2020; Johnson et al., 2020; Kortenkamp, 2020; Yokel, 2020; Palermo et al., 2021; Silva da et al., 2021; Myden et al., 2022; Pedersen et al., 2022; Kose et al., 2023). A partial AOP for decreased gonadotropin-releasing hormone (GnRH) expression in hypothalamus leading to reduced testosterone production has been postulated (Lu et al., 2023). There are also published putative AOPs for female reproductive toxicity, including disrupted folliculogenesis and follicle maturation, decreased ovulation, disrupted puberty onset in girls, uterine tumours, and breast cancer causally linked to disrupted hypothalamic–pituitary–gonadal (HPG) axis signalling, ER activation, estrogen metabolism, and aromatase inhibition (Del’haye et al., 2022; Franssen et al., 2022; Hernandez-Jerez et al., 2023; Johansson et al., 2020; Myden et al., 2022; Wikoff et al., 2016). Apart from the challenge of finding AOPs published in the literature, such AOPs cannot be included in the AOPN using the computational automated process and must be manually added.

In conclusion, the AOPN developed here summarises AOPs for mammalian reproductive toxicity via disruption of the EAS modalities currently available in the wiki. It will form the basis for further research and AOP development, including deeper analyses of the subnetworks and core KE(R)s to identify specific areas of prioritization. Such future activities entail, for example, identifying central KERs and developing them further to increase the quantitative understanding, as well as analysing the extent to which EAS-mediated effects relevant for regulatory ED assessment are covered by current AOPs to inform further AOP development. The AOPN is being further refined and applied in ongoing projects with the aim to provide a more complete AOPN and practical examples showcasing how this methodology can be used to support the identification and assessment of EDs.

## Data Availability

The original contributions presented in the study are included in the article/Supplementary Material, further inquiries can be directed to the corresponding author.
