# Supplementary figures and images for: Building an adverse outcome pathway network for estrogen-, androgen- and steroidogenesis-mediated reproductive toxicity
Source: Front Toxicol. 2024 Mar 26;6:1357717. doi: 10.3389/ftox.2024.1357717 (PMC11005472; doi:10.3389/ftox.2024.1357717)

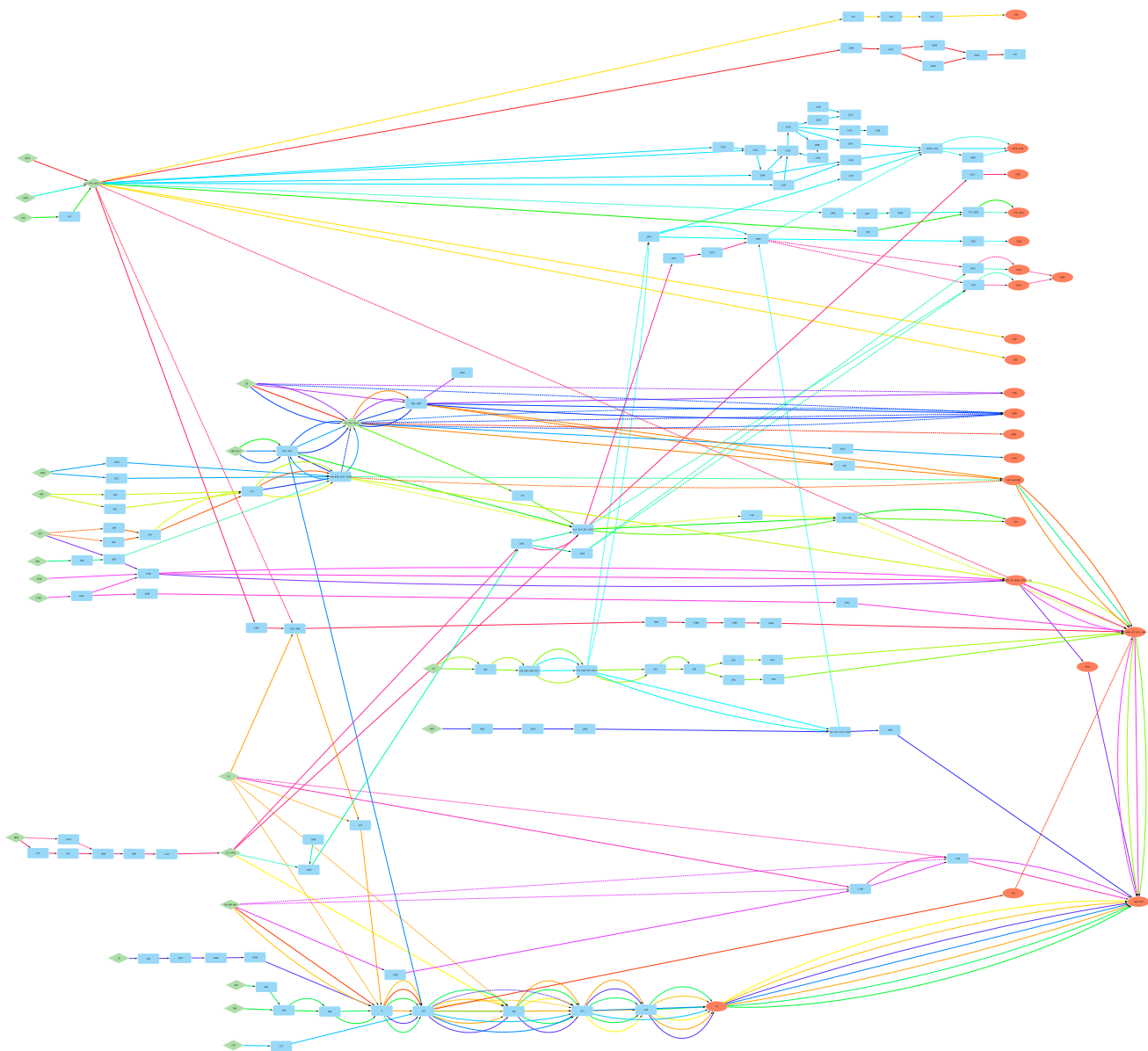

Supplement: Supplementary file 1 [file Image2.PDF]

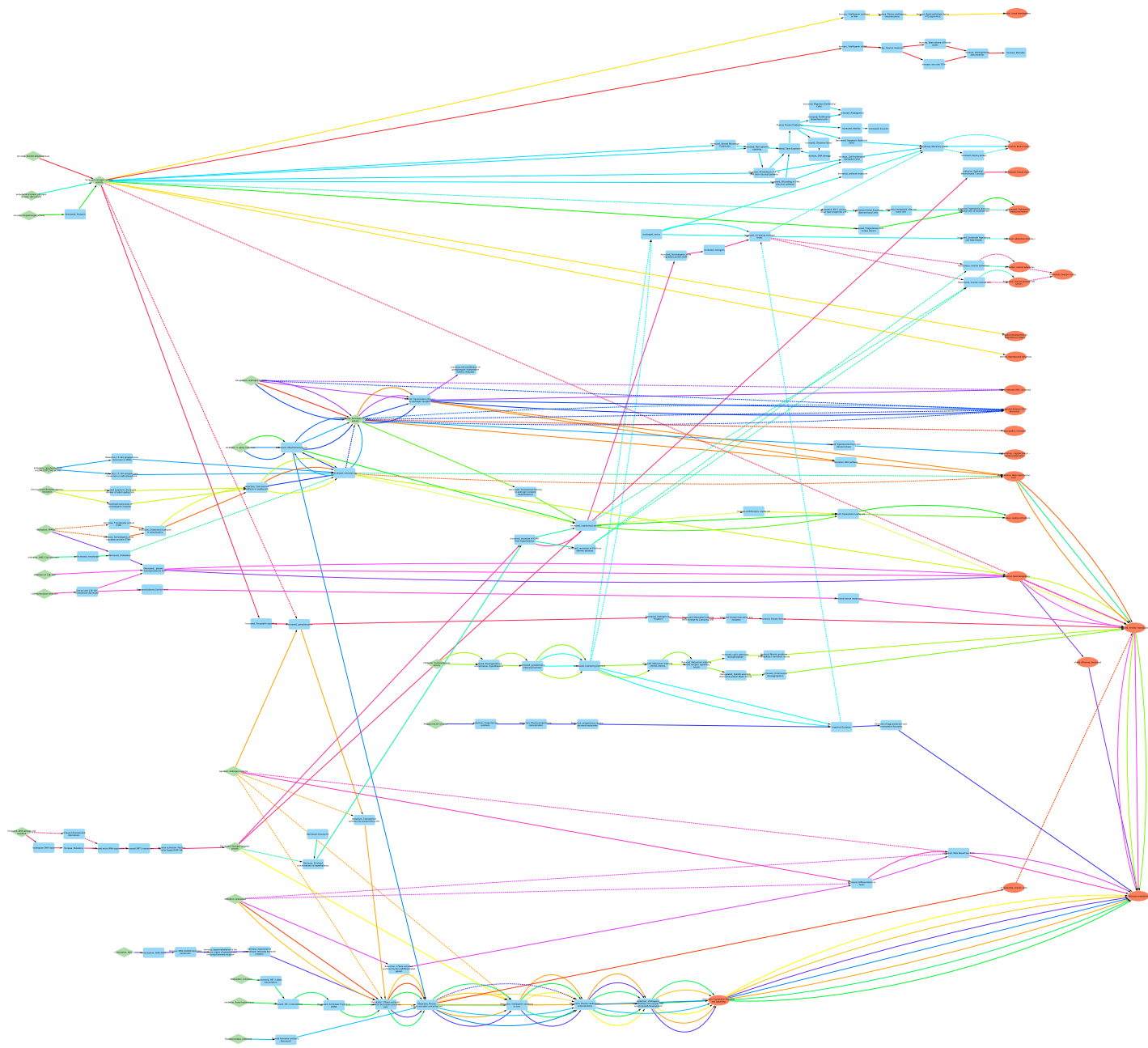

Supplement: Supplementary file 4 [file Image1.PDF]
