# Supplementary material for: Building an adverse outcome pathway network for estrogen-, androgen- and steroidogenesis-mediated reproductive toxicity
Source: Front Toxicol. 2024 Mar 26;6:1357717. doi: 10.3389/ftox.2024.1357717 (PMC11005472; doi:10.3389/ftox.2024.1357717)
Supplement: Supplementary file 2 [file DataSheet1.PDF]

Supplementary Material

Building an adverse outcome pathway network for estrogen-, androgen- and steroidogenesis-mediated reproductive toxicity.

Johanna Zilliacus[1], Monica K. Draskau[2], Hanna K.L. Johansson[2], Terje Svingen[2] and Anna Beronius[1]*

[1]Institute of Environmental Medicine, Karolinska Institutet, Sweden

[2]National Food Institute, Technical University of Denmark, Denmark

*** Correspondence:**
Anna Beronius
anna.beronius@ki.se

1 Supplementary Data

Supplementary Data sheet S1 - Parameters and key terms, Cytoscape input-before combination of KEs, Cytoscape final input, AOPs, KEs, KERs, Core KEs, Core KER

2 Supplementary Figures and Tables

Supplementary Figure S1 – High resolution AOP network with KE titles

Supplementary Figure S2 – High resolution AOP network with KE IDs
